# Epithelial-to-mesenchymal transition is an active process in the large airways of patients with asthma-COPD overlap and partially abrogated by inhaled corticosteroid treatment: a bronchoscopy endobronchial biopsy study

**DOI:** 10.3389/fimmu.2025.1531279

**Published:** 2025-05-22

**Authors:** Surajit Dey, Wenying Lu, Prabuddha S. Pathinayake, Maddison Waters, Greg Haug, Josie Larby, Heinrich C. Weber, Peter A. B. Wark, Mathew Suji Eapen, Sukhwinder Singh Sohal

**Affiliations:** ^1^ Respiratory Translational Research Group, Department of Laboratory Medicine, School of Health Sciences, College of Health and Medicine, University of Tasmania, Launceston, TAS, Australia; ^2^ Immune Health Program, Hunter Medical Research Institute, University of Newcastle, New Lambton Heights, Australia; ^3^ Department of Respiratory Medicine, Launceston General Hospital, Launceston, Australia; ^4^ Department of Respiratory Medicine, Tasmanian Health Services (THS), North-West Hospital, Burnie, TAS, Australia; ^5^ Department of Respiratory Medicine, Monash University, Melbourne, Australia

**Keywords:** asthma-COPD overlap (ACO), COPD - chronic obstructive pulmonary disease, smoking, fibrosis, ICS - inhaled corticosteroids, histopathology, diagnosis, EMT - epithelial to mesenchymal transformation

## Abstract

**Introduction:**

Asthma and chronic obstructive pulmonary disease (COPD) overlap (ACO) is a term used to describe a patient with coexisting clinical features of asthma and COPD. We have previously reported that epithelial to mesenchymal transition (EMT) is active in the lungs of patients with COPD however, EMT in ACO remains an unexplored area. We hypothesize that EMT is an active process in ACO.

**Methods:**

In this cross-sectional study, large airway endobronchial biopsy (EBB) tissues from patients with asthma (14), COPD (22), current (CS) and ex-smokers (ES), and ACO (12) were immunohistochemically stained for EMT markers (E and N cadherin, vimentin, S100A4, and Collagen IV) and compared with 12 current smokers with normal lung function (NLFS) and 10 non-smoking healthy control (HC) subjects. In addition, air-liquid interface (ALI) cell cultures were performed and cells from patients with ACO and HC were treated with TGF-β, IL-13 and cigarette smoke extract (CSE). Later cells from ALI cultures were lysed for Immunoblotting. Immunostained tissues were enumerated for percent expression of E and N-Cadherin in the epithelium, vimentin and S100A4 positive cells both in the epithelium and reticular basement membrane (RBM). Additionally, the degree of RBM fragmentation was evaluated, a key tissue structural marker of EMT.

**Results:**

Compared to healthy controls and asthmatics, ACO had the greatest fragmentation of RBM (P < 0.01). ACO also had substantially decreased percentage expression of E-cadherin (P <0.01), increase percentage of N-cadherin expression, and higher vimentin and S100A4 positive basal cells, in comparison to healthy controls. In the RBM of ACO, S100A4 positive cells (P <0.05) and Vimentin-positive cells were markedly higher in comparison to HC. Similar changes were observed with western blots in response to Th-2 cytokine IL-13, CSE and EMT activator TGF-β.

**Conclusions:**

These data are suggestive of active EMT in ACO. Additionally, 50% of the patients with ACO were on 800 mcg/day inhaled corticosteroid (ICS) treatment which may have abrogated some EMT activity; however, it suggests protective effects of ICS as we previously reported in COPD. Studies with larger cohorts are needed to further confirm ICS effects in ACO.

## Introduction

Asthma and COPD are chronic respiratory diseases affecting millions of people worldwide (1). Associations between these two diseases have been acknowledged over the previous decades and recently received an official designation of ACO (1). If asthma and COPD are considered diseases with distinct pathologies, one might conceive that the ACO is also a discrete disease entity with its own pathology as determined by the genetic and environmental determinants. ACO may also simply be a manifestation of continued airway disease, positioned at the fulcrum of asthma and COPD phenotypes, showing features associated with both conditions (2). Although this debate is never-ending, ACO is nowadays considered a treatable trait for better patient management (1). Nonetheless, the underlying cellular and molecular mechanisms for the development of ACO require a detailed investigation to understand the involvement of undoubtedly complex mechanisms.

Epithelial to mesenchymal transition (EMT) is an important cellular program during which the epithelial cells lose their adhesive property and attain mesenchymal phenotype with a more migratory property due to cell signals triggered from the cellular microenvironment (3, 4). Generally, the cells of the epithelial layer of tissue show apical-basal polarity and are held tightly by the tight, adheren junctions such as E-cadherin and chained to the reticular basement membrane (RBM) by hemidesmosomes. When EMT is activated, E-cadherin expression is suppressed, and the tight epithelium junction is loosened, leading to acquisition of mesenchymal proteins. The cells then take up the mesenchymal phenotype with fibroblast-like morphology and express markers such as N-cadherin, vimentin, or S100A4. The EMT has consequences to wound healing, tissue regeneration, organ fibrosis and cancer.

EMT is the core pathophysiological process in COPD (5–7). In smoking-associated COPD, recent research highlighted that EMT is linked to airway remodelling, airway fibrosis, cancer and subsequent airflow obstruction (8–11). Several studies including from our research group have reported active EMT in patients with COPD but there is no evidence from the lungs of patients with ACO. The presence of smoking and chronic inflammation in ACO is sufficient to inflict epithelial injury. Lately, we reported thickened RBM and abundant RBM cells in the airways of patients with ACO (12). Furthermore, our recent report indicated a hyper vascular RBM in patients with ACO, suggesting a possible angiogenesis in these patients, the so-called Type III EMT (1, 9, 13). Collectively, we hypothesize that the EMT process may be active in the airways of patients with ACO.

## Methods

To evaluate the above hypothesis, we analyzed the large airway biopsies tissues of patients with ACO, which were compared against the HC, asthma, COPD-ES and CS, and NLFS for the EMT markers E- and N-cadherins, vimentin, and S100A4. In addition, the degree of RBM fragmentation, a hallmark of EMT activity (8) was measured and compared among the groups.

### Participant demographics

A total of 70 large airway endobronchial biopsy (EBB) samples were collected from participants (12 ACO, 14 asthmatics, 10 COPD ex-smokers (ES), 12 COPD current-smokers (CS), 12 NLFS, and 10 HC) and were obtained from the Tasmanian Respiratory Tissue bank and Newcastle biobanks (Tasmanian Health and Medical Human Research Ethics Committee, ethics IDs: H0013051; the Hunter New England Human Research Ethics Committee reference no: 05/08/10/3.09). The details of tissue collection from the research volunteers were reported in our earlier publications (12–14). Among the participants with ACO, 7 were classified as GOLD stage I and II and 1 as GOLD stage III COPD considering their lung function, and 4 were classified as having severe asthma. The majority of participants with ACO were ex-smokers. Participants with COPD were of mild to moderate COPD (GOLD stage I and II). Fifty percent of the patients with ACO and asthma were on inhaled corticosteroids (800/750 - mcg/day). All participants with asthma and HC were non-smokers. None of the HC subjects had a history of respiratory illness. A summary of the participant demographics is presented in **Table 1**.

**Table 1:** Patients/subject demographics.

Parameters/Groups	HC	ACO	Asthma	COPD-ES	COPD-CS	NLFS
Subjects	10	12	14	10	12	12
Age (years)	61 (25–68)	70 (52–77)	62 (26-81)	67 (46-78)	65.5 (51-78)	56.5 (41-72)
Smoking History (pack-year)	0	22.5 (15-103)	0	36 (22-105)	34.8 (10-114)	31.5 (20-75)
ICS treatment (n)	N/A	6	6	N/A	N/A	N/A
ICS dose	N/A	800 mcg/day	750 mcg/day	N/A	N/A	N/A
GINA Diagnosis Mild persistent/ Moderate/Severe (n)	N/A	0/0/4	5/1/8	N/A	N/A	N/A
Gold Diagnosis Stage I &II/Stage III (n)	N/A	7/1	N/A	10/0	12/0	N/A
%FEV_1_	93 (75-114)	58 (35-96)	81.5 (48-108)	84.5 (54-113)	69 (49-92)	94 (79-113)
%FEV_1_/FVC	83 (73-86)	65.5 (31-84)	74 (52-90)	63.2 (55-69)	63.6 (50-75.3)	78 (70-85)

Data expressed as median and range.

ACO, asthma COPD overlap; COPD, chronic obstructive pulmonary disease; COPD-CS, COPD current smokers; COPD-ES, COPD ex-smokers; FEV1, forced expiratory volume in 1 second; FVC, forced vital capacity; GINA, The Global Initiative for Asthma; GOLD, The Global Initiative for Chronic Obstructive Lung Disease; HC, healthy control; n, number of subjects/patients; N/A, not applicable; NLFS, normal lung function smokers; ICS, inhaled corticosteroids.

### Immunohistochemical staining

The formalin-fixed, paraffin-embedded biopsy tissue blocks were sectioned at 3 µm and placed on positively charged glass slides. These sections were dried overnight. Paraffin sections were then dewaxed in xylene, and rehydrated using graded ethanol, followed by a wash in distilled water. Heat-Induced epitope retrieval was conducted in a decloaking chamber (Biocare Medical) using low pH Dako target retrieval solution (Cat#.S1699). The sections were then treated with 3% hydrogen peroxide (H_2_O_2_) in distilled water (v/v) to block endogenous peroxidase activity. Primary antibodies were applied to the tissue sections as follows: mouse monoclonal E-cadherin (1:200 dilution, ab1416, Abcam, Victoria, Australia), mouse monoclonal N-cadherin (1:150 dilution, ab98952, Abcam, Victoria, Australia), rabbit polyclonal S100A4 (1:1000, A5114, Dako, Victoria, Australia), mouse monoclonal Vimentin (1:200, M7020 Dako, Victoria, Australia), rabbit polyclonal Collagen IV (1:350, ab6586, Abcam, Victoria, Australia), incubated at room temperature. Bound antibodies are then elaborated using polymer enzyme backbone conjugated secondary antibody (Dako EnVision, K5007) and visualised using diaminobenzidine (DAB) chromogen (Dako EnVision, K7005). Harris hematoxylin was used for the nuclear staining. Before performing immunostaining, optimisation of manufacturer-specific immunohistochemical methods was conducted. We have published with these methods before (15–18).

### Quantification of immuno-stained biopsy tissue

**Figure 1:**
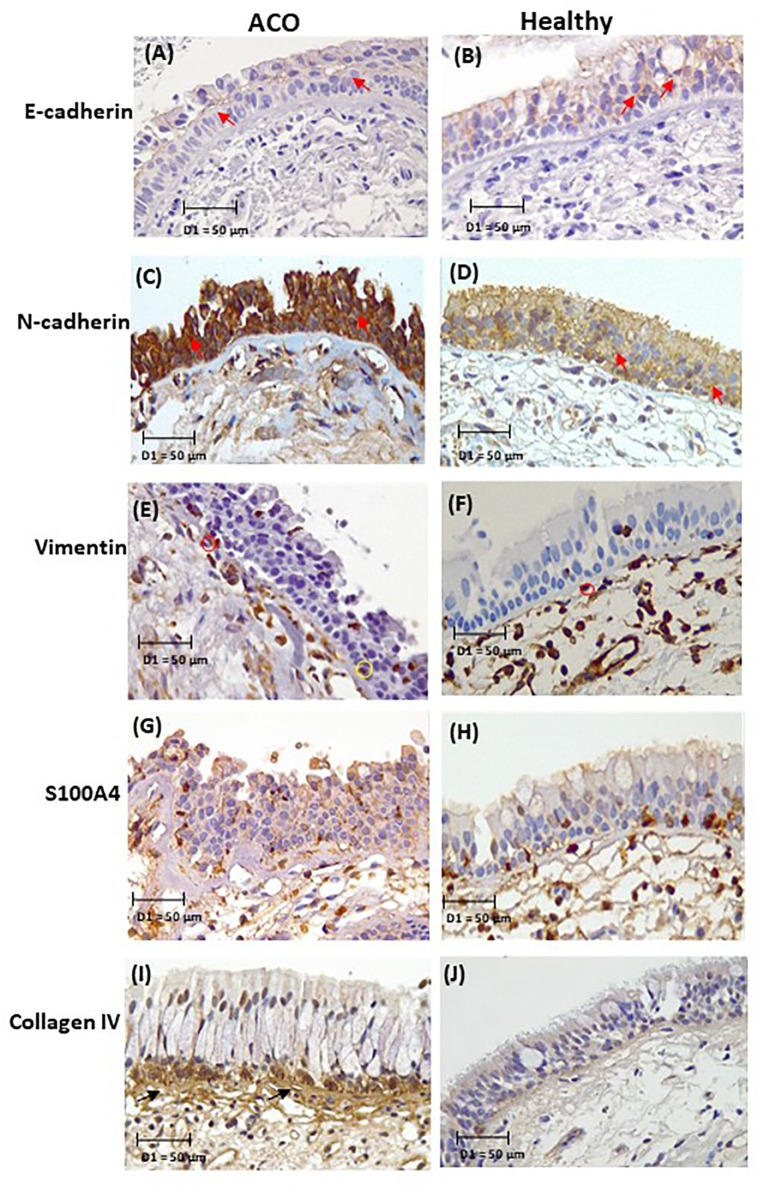
Representative micrographs of EMT markers in ACO and HC: E-cadherin **(A, B)**, N-cadherin **(C, D)**, vimentin **(E, F)**, S100A4 **(G, H)**, and collagen IV **(I, J)**. Tissue micrograph showing the junctional E-cadherin and N cadherin “red arrow”, marker positive basal cells “yellow circle”, RBM cells “red circle”, and RBM fragments “black arrow”.

Computer-assisted image analysis was performed with a Leica DM 500 microscope (Leica Microsystems, Germany) and a Leica ICC50W camera. Tissues with visible epithelium, RBM, and lamina propria (LP) were selected for image analysis. The images of the entire tissue area, including epithelium, RBM and LP, were captured at 40X brightfield, avoiding the overlapping area between images. Five images were randomly chosen for percentage stain and cell counts. For E- and N-cadherin, the percent staining expression was measured in the epithelium. For the mesenchymal markers, the marker-positive basal cells (brown) in the epithelial and the marker-positive cells in RBM were counted (**Figures 1E, F**), presented as cells per mm of RBM length. The observer was blinded to patient and diagnosis. The representative tissue micrographs of EMT markers in ACO and HC are provided in **Figure 1**.

Length of fragments was measured from Collagen IV-positive tissues which separated the fragments from vessels (**Figure 1I**). Fragmentation of the RBM included pieces apparently hanging off and fully separated from the rest and clefts within the RBM. The total length of splits was added and divided by the length of RBM. Splits that were parallel to the RBM were also included in the measurement. Image analyses were performed using the image analysis software Image-Pro Plus 7.0.

### Cigarette smoke extraction

Cigarette smoke extract (CSE) was prepared by bubbling smoke from one filter-less Kentucky research cigarette, 3R4F, which contains 9.5 mg of tar and 0.8 mg of nicotine, through 10 mL of Bronchial Epithelial Basal Medium (BEBM, Lonza) at a rate of one cigarette every 5 minutes (19). This was used immediately in subsequent cell culture experiments by diluting it with BEBM media to achieve a concentration of 1%. We previously determined that this concentration of CSE, assessed via dose-response curves, causes minimal toxicity to the cells while still inducing an immune response. All cells were grown at 37°C with 5% CO2 air.

### Cell culture and treatment

Primary bronchial epithelial cells (pBECs) were collected from healthy (n=3) and ACO (n=3) subjects via bronchoscopy. pBECs from the healthy control and ACO groups were cultured in separate culture plates as air-liquid interface cultures. In each plate, transwells were spaced out one well apart to minimize cross-contamination. Treatments were added carefully into the basal compartment. A new cell culture pipette tip was used on each treatment for each well on every media change. Primary cells were grown in complete BEGM (Lonza) with growth factor supplements in a submerged monolayer culture. Once confluent, cells were trypsinised using a 1:10 dilution of the standard 0.25% trypsin-EDTA solution (Sigma), and once the cells are dissociated, FBS was used to neutralise and then seeded at 2 × 10^5^ cells in trans-wells in a 12-well plate (Corning) with PneumaCult™-Ex Plus medium containing amphotericin B (final concentration 250 μg/ml), and 2% penicillin-streptomycin until confluent (at least 3 days in both apical and basal compartments) (20–24). Once confluent, apical media was removed and the basal media was replaced with PneumaCult™-ALI medium containing amphotericin B (final concentration 250 μg/ml), and 2% penicillin-streptomycin and maintained at the air-liquid interface (ALI) phase for 21 days until fully differentiated. Fully differentiated ALI wells at day 21 were basolaterally treated with EMT drivers (25) TGF-β (5ng/mL) and interleukin (IL)-13 (10ng/mL) (26) for 10 days or with 1% cigarette smoke extract (CSE) for 2 days. At the end of the treatment, cells were harvested for RNA or protein using appropriate lysis buffers.

### Immunoblotting

Air-liquid interface (ALI) samples were lysed with RIPA buffer containing protease inhibitor (Sigma-Aldrich, Australia) for 20 minutes at 4°C and were assessed for total protein concentrations using pierce™ BCA protein assay kits (Thermofisher Scientific, Australia) (27, 28). A total of 5ug protein was loaded into activated Mini-PROTEAN^®^ TGX Stain-Free™ precast gels (Bio-Rad laboratories Australia) and SDS-PAGE separation was performed. Standard immunoblot was performed against E-cadherin (14472S, Cell signalling), N-cadherin (ab18203, Abcam), and Vimentin (ab92547, Abcam) as described previously (29). β-actin (ab8227, Abcam) was used as the loading control. Protein bands were detected using SuperSignal West Femto Maximum Sensitivity Substrate reagents (ThermoFisher Scientific Inc.) on the ChemiDoc MP Imaging System (Bio-Rad Laboratories). Protein band density was measured using Image J software and normalized to the loading control (β-actin) level. The relative expression level of protein was calculated and graphed using GraphPad Prism 9.0 software.

### Statistical analysis

Following the data distribution check using D’Agostino & Pearson test, intra- and inter-group variances were analysed using one-way ANOVA or nonparametric with multiple comparisons using uncorrected Dunn’s test. Unless otherwise mentioned, the results are reported as median and range. Univariate Spearman *r* was used for correlation analysis. Furthermore, the effect of ICS on the EMT markers was explored using a nonparametric test. A *P* value of <0.05 was considered significant. All analyses were done using GraphPad Prism v9 (San Diego, CA, USA).

## Results

### Expression of EMT markers in the epithelium

**Figure 2:**
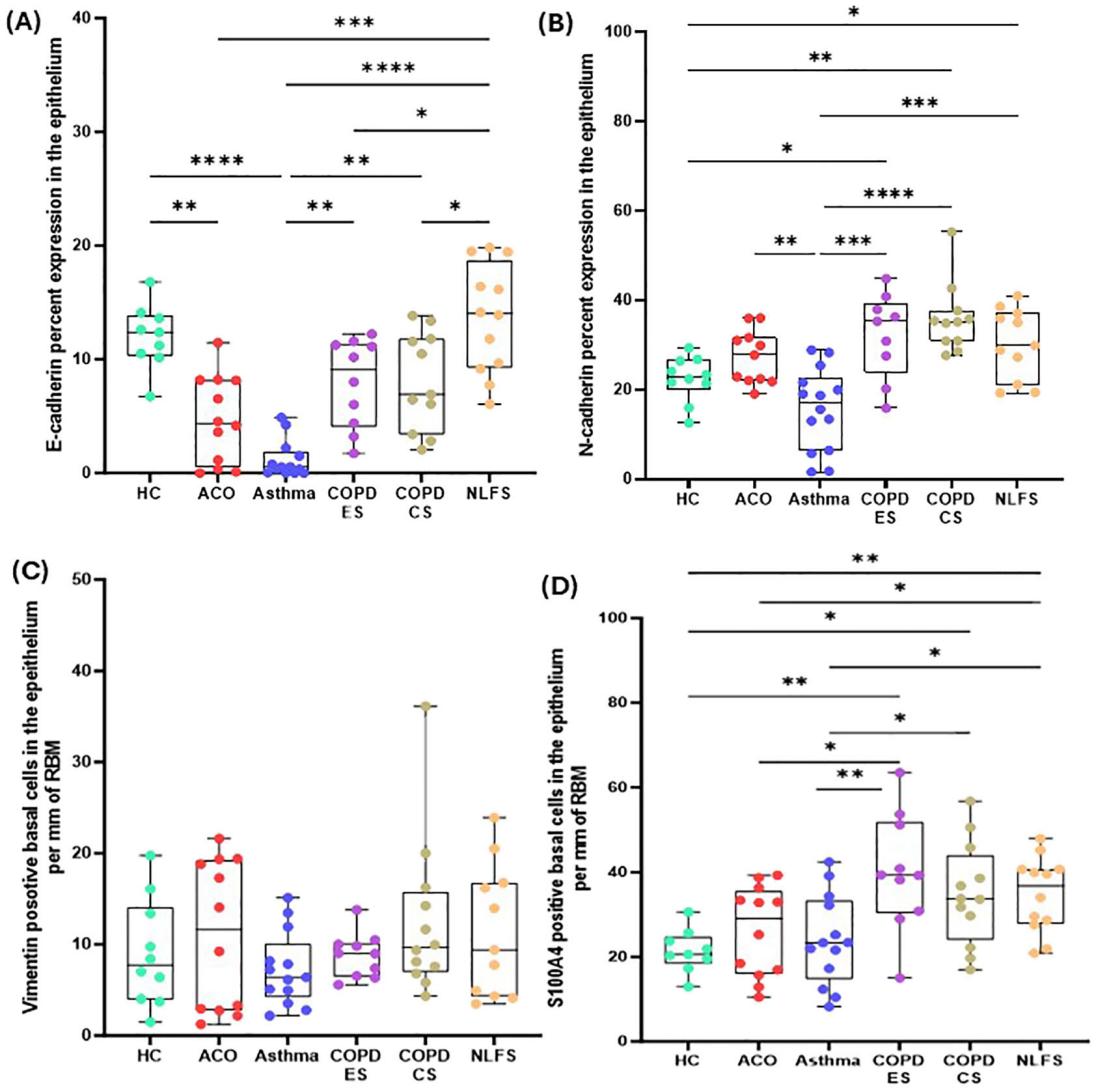
Box plots showing percent expression of E-cadherin **(A)**, N-cadherin **(B)**, vimentin-positive basal cells per mm of reticular basement membrane (RBM) **(C)**, S100A-positive basal cells per mm RBM **(D)** in HC, ACO, asthma, COPD-ES and COPD-CS, and NLFS. The horizontal line inside each box represents the median; the top and bottom of each box represent the upper and lower quartiles, respectively; and the whiskers represent extreme values. *P* value representation * <0.05, ** <0.01, *** <0.001, **** <0.0001.

The epithelial marker E-cadherin percentage expression in large airway epithelium decreased across all pathological groups (**Figure 2**). In contrast, the mesenchymal markers N-cadherin percent expression, vimentin, and S100A4 positive cell counts generally increased in all pathological groups except in the patients with asthma. E-cadherin percent expression in patients with ACO was substantially decreased as compared to the HC (*P <*0.01) and NLFS (*P <*0.001) groups and tended to be lower than the COPD groups (**Figure 2A**). In addition, a notable decrease in E-cadherin expression was also noted in COPD-ES (*P <*0.05) and COPD-CS (*P <*0.05) groups as compared to NLFS; however, compared to HC the decrease in percent expression COPD-ES (*P* = 0.1021) and CS (*P* = 0.1178) was not statistically significant.

**Figure 3:**
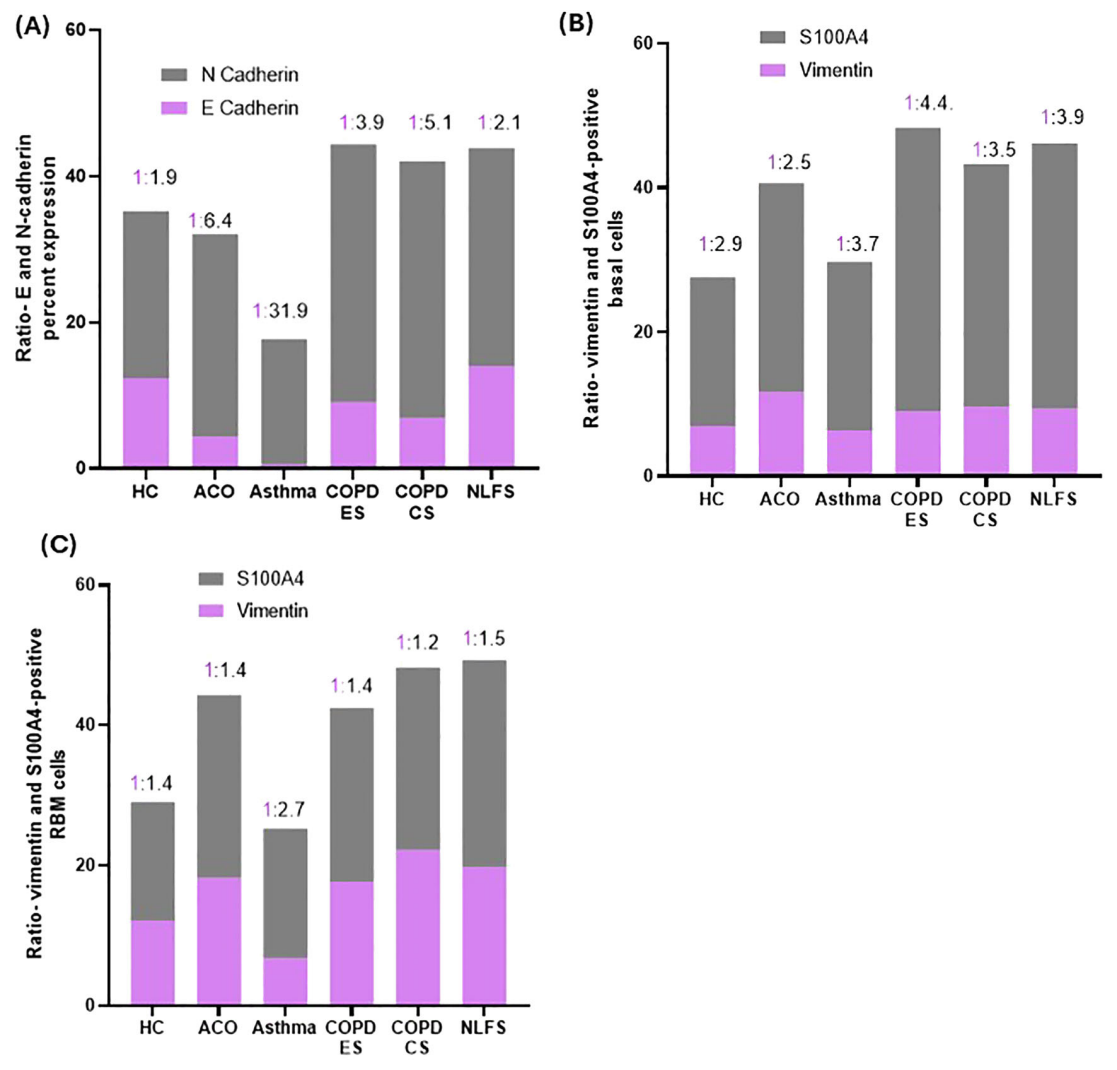
Column plots showing the ratios of percent expression of E and N-cadherin **(A)**, vimentin and S100A4-positive basal cells **(B)** and RBM cells **(C)** in HC, ACO, asthma, COPD-ES and COPD-CS, and NLFS.

N-cadherin percent expression in the ACO group appeared to be higher (P = 0.1608) than in the HC group and tended to be lower than in the COPD groups (ES, *P* = 0.3107; CS, *P* = 0.0702) and NLFS (*P* = 0.5487) (**Figure 2B**). Compared to asthma, the percent expression of N-cadherin increased markedly in ACO (*P <*0.01). Although the percent expression of N-cadherin remained similar in between COPD groups, we further noticed a substantially higher expression of N-cadherin in COPD-ES and CS groups than in the HC (*P <*001 and <0.05, respectively) and asthma (*P <*0.001 and <0.0001, respectively). The ratio of E and N cadherin percent expression indicated a dominance of N cadherin, a mesenchymal marker as compared to HC which had near similar proportion of E and N cadherin percent expression (**Figure 3A**).

The number of vimentin-positive basal cells was highest in the patients with ACO followed by the patients with COPD (**Table 2**, **Figure 2C**). However, compared to HC, the differences were statistically not significant (ACO, *P* = 0.6909; COPD-ES, *P* = 0.7259; CS, *P* = 0.2686). In asthma, vimentin-positive basal cells were similar to the HC (*P* = 0.5150).

**Table 2:** EMT markers and degree of fragmentation among HC, ACO, asthma, COPD-ES, COPD-CS, and NLFS.

EMT markers/Groups	HC	ACO	Asthma	COPD-ES	COPD-CS	NLFS
E-cadherin						
Epithelial E-cadherin (% expression)	12.4 (6.7-16.8)	4.4 (0-11.5)	0.52 (0-4.9)	9.1 (1.7-12.2)	6.9 (2.1-13.8)	14.0 (6.1-19.8)
N-cadherin						
Epithelial N-cadherin (% expression)	22.9 (12.6-29.3)	27.8 (19.1-36.0)	17.2 (1.6-28.9)	35.3 (15.9-44.8)	35.1 (27.7-55.3)	29.9 (19.3-40.9)
Vimentin						
Basal cells/mm of RBM	7.7 (1.5-19.8)	11.7 (1.2-21.6)	6.4 (2.2-15.1)	9.0 (5.6-13.8)	9.7 (4.4-36.1)	9.4 (3.5-23.9)
RBM cells/mm of RBM	12.2 (3.4-20.4)	18.2 (5.6-38.2)	6.8 (0-32.0)	17.7 (10.8-28.2)	22.23 (8.7-27.6)	19.77 (7.9-33.0)
S100A4						
Basal cells/mm of RBM	20.6 (12.0-30.6)	29.0 (10.5-39.3)	23.4 (8.2-42.4)	39.3 (15.0-63.5)	33.7 (17.0-56.7)	36.8 (20.9-47.9)
RBM cells/mm of RBM	16.9 (8.4-26.7)	26.1 (12.2-35.0)	18.5 (5.2-41.1)	24.8 (15.5-36.1)	26.0 (16.3-46.9)	29.6 (15.7-39.2)
Collagen IV						
Degree of RBM fragmentation	7.9 (2.6-12.0)	17.3 (7.0-52.1)	7.0 (1.2-26.1)	10.5 (0.95-50.9)	13.9 (6.5-27.3)	12.9 (6.7-21.3)

Data presented as median (minimum – maximum).

ACO, asthma COPD overlap; COPD, chronic obstructive pulmonary disease; COPD-CS, COPD current smokers; COPD-ES, COPD ex-smokers; RBM, reticular basement membrane; LP, lamina propria; NLFS, normal lung function smokers.

The number of S100A4-positive basal cells was higher in all pathological groups (**Figure 2D**). Although in the ACO group S100A4 positive basal cells appeared to be higher than the HC (*P* = 0.3376) and asthma group (*P* = 0.7417), we noted significantly lower basal cells in ACO than in COPD-ES (*P <*0.01) and NLFS (*P <*0.05) groups. S100A4 positive basal cells were tended to be lower in ACO than in COPD-CS group (*P* = 0.1029). Furthermore, the S100A4-positive basal cells were markedly enhanced in both COPD-ES and CS groups as compared to the HC (*P <*0.01 and <0.05, respectively) and asthma (*P <*0.01 and <0.5, respectively). S100A4-positive basal cells in asthma were essentially normal. When we plotted the ratio of vimentin and S100A4 positive basal cells, we noted a higher proportion of S100A4 positive cells across all groups (**Figure 3B**).

### The degree of reticular basement membrane fragmentation (RBM, key EMT tissue hallmark)

**Figure 4:**
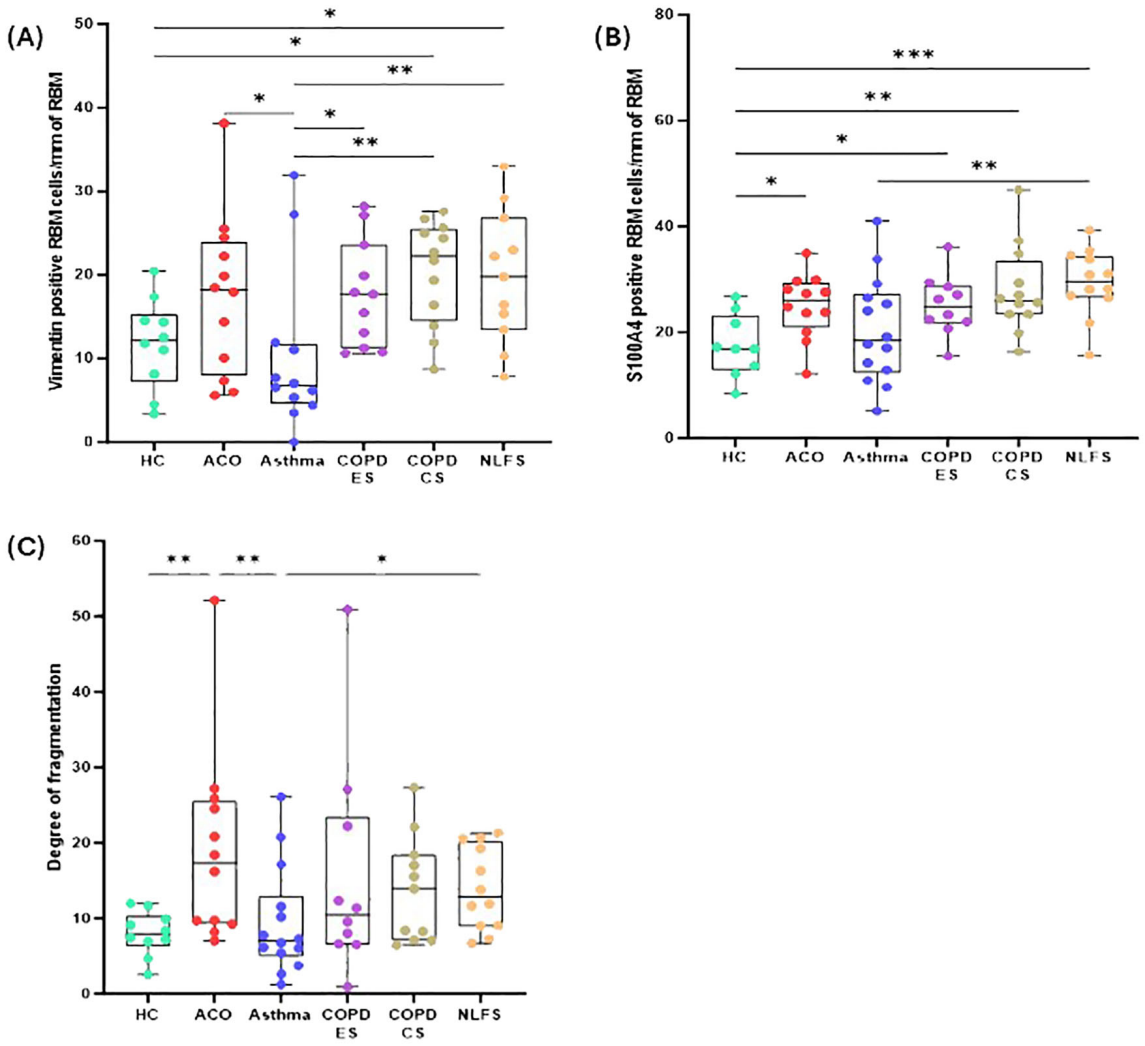
Box plots showing vimentin-positive cells in of reticular basement membrane (RBM) per mm of RBM **(A)**, S100A-positive cells in RBM per mm of RBM **(B)**, degree of fragmentation **(C)** in HC, ACO, asthma, COPD-ES and COPD-CS, and NLFS. The horizontal line inside each box represents the median; the top and bottom of each box represent the upper and lower quartiles, respectively; and the whiskers represent extreme values. P value representation * <0.05, ** <0.01, *** <0.001.

The degree of fragmentation was greatest in patients with ACO followed by COPD-CS and ES, and lowest in the asthma group (**Figure 4C**). Enhanced fragmentation in the ACO group was statistically significant than the HC (*P <*0.01) and asthma (*P <*0.01). Although the fragmentation in the ACO group appeared to be higher than the COPD-CS (*P* = 0.3215) and ES (P= 0.2017), the difference was not statistically significant. Furthermore, the degree of fragmentation was substantially higher (*P <*0.05) in COPD-CS than in the asthma group and tended to be higher than in the HC (P=0.1086). In the COPD-ES group, the fragmentation tended to be higher (*P*= 0.2039) than the HC and lower than the COPD-CS group (*P* = 0.7612). The RBM fragmentation in patients with asthma was very similar to HC.

### Expression of mesenchymal markers in the RBM

In general, similar to the epithelial basal cells, both vimentin and S100A4 positive cells (**Figures 4A, B**) in the RBM of pathological groups were elevated except in patients with asthma.

The vimentin-positive cells appeared to be elevated in the RBM of ACO as compared to HC although the difference was not statistically (P = 0.1610); however, as compared to asthma the increase in vimentin-positive cells was notable (*P <*0.05). The number of vimentin-positive cells in patients with ACO was similar to that of COPD-ES (*P* = 0.7397) and NLFS (*P* = 0.4331) groups. The COPD-CS group also had high vimentin-positive RBM cells, compared to HC and asthma groups (*P <*0.05 for both). On the other hand, the vimentin-positive cells in asthma tended to be lower than HC (P = 0.5641). Similar to the epithelial region, the ratio of vimentin and S100A4 RBM cells suggested a higher proportion of S100A4 positive cells across all groups (**Figure 3C**).

In the RBM, a significantly higher number of cells were stained for S100A4 in ACO (*P <*0.05), COPD-ES (*P <*0.05), COPD-CS (*P <*0.01), and NLFS (*P <*0.001) as compared to HC. The S100A4 stained cells remained comparable among patients with ACO, COPD-ES, COPD-CS, and NLFS. The S100A4 stained RBM cells in asthma remained similar to HC.

### Exploratory analysis

**Figure 5:**
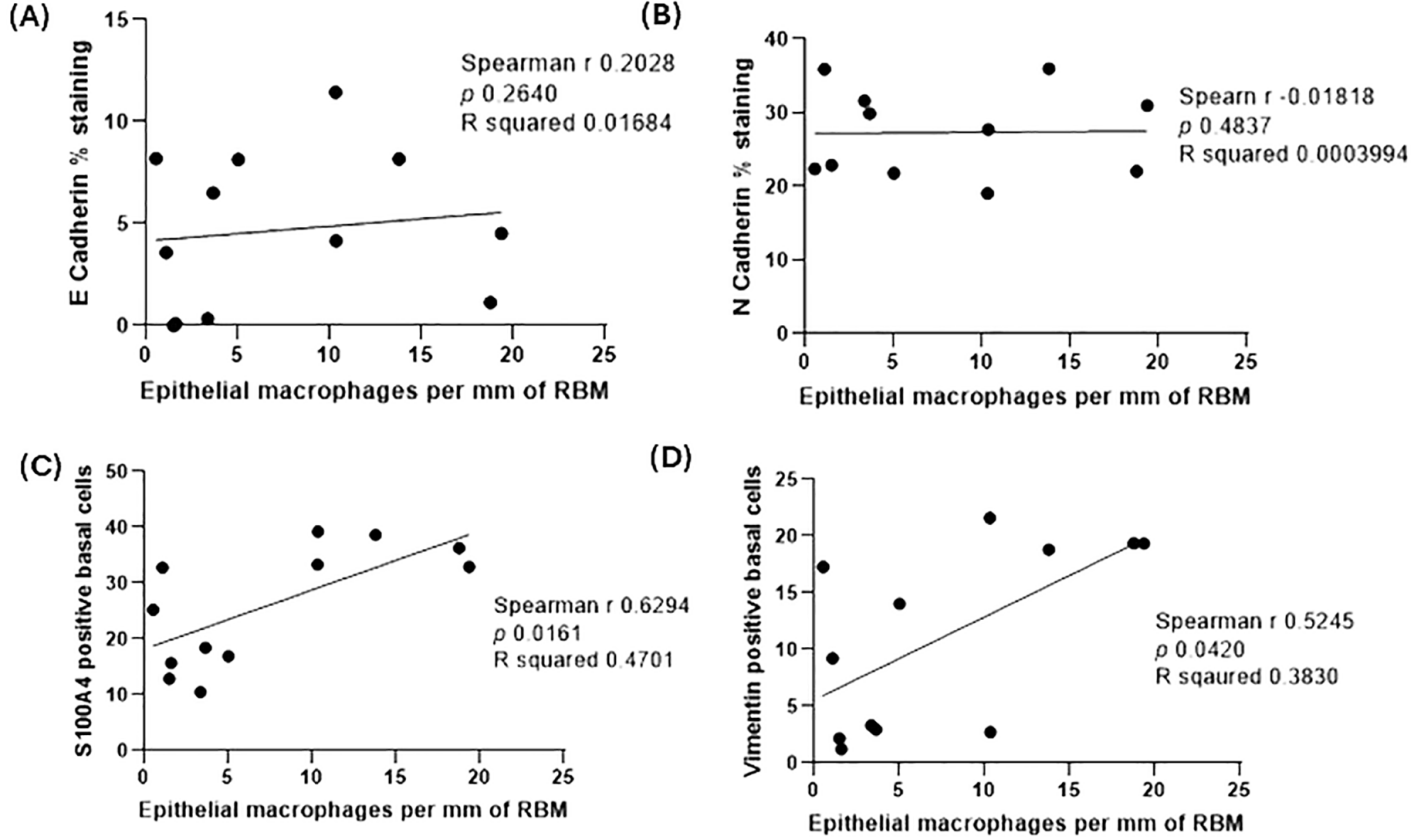
Spearman correlation analysis between E-cadherin percent expression and epithelial macrophages per mm of the reticular basement membrane (RBM) from samples of ACO **(A)**, N-cadherin percent expression and epithelial macrophages per mm of RBM from samples of ACO **(B)**, S100A4 positive basal cells and epithelial macrophages per mm of RBM from samples of ACO **(C)**, and vimentin positive basal cells and epithelial macrophages per mm of RBM **(D)** from samples of ACO.

**Figure 6:**
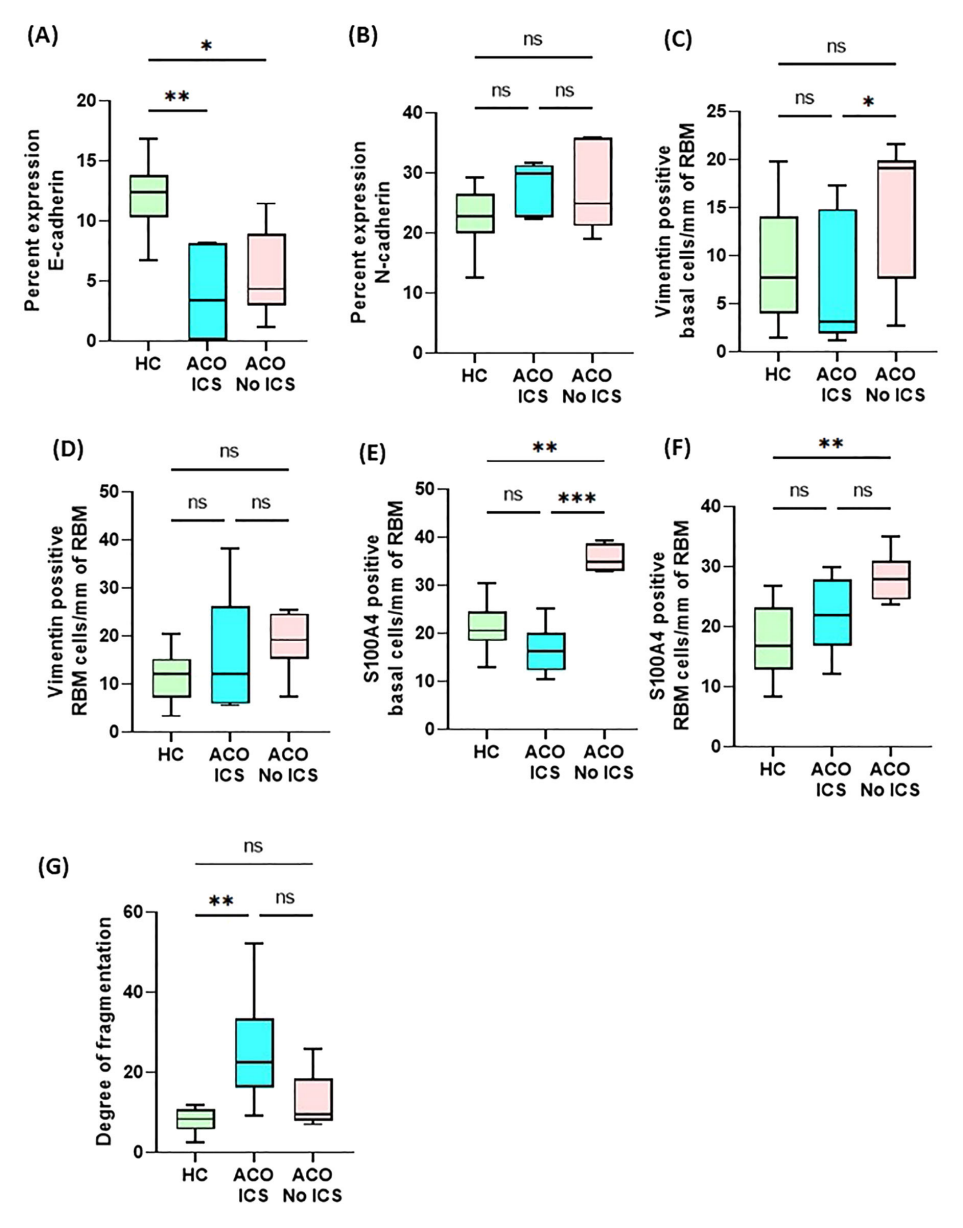
Box plots showing percent expression of E-cadherin **(A)**, N-cadherin **(B)**, vimentin-positive basal cells/mm of reticular basement membrane (RBM) **(C)**, vimentin-positive RBM cells/mm of RBM **(D)**, S100A4-positive basal cells/mm of RBM **(E)**, S100A4-positive RBM cells/mm of RBM **(F)**, and degree of fragmentation **(G)** in HC and asthma, and ACO with and without inhaled corticosteroids (ICS). The horizontal line inside each box represents the median; the top and bottom of each box represent the upper and lower quartiles, respectively; and the whiskers represent extreme values. ANOVA P value representation * <0.05, ** <0.01, and *** <0.001.

We performed an array of correlation analyses using the FEV_1_/FVC ratio, smoking history with EMT markers from samples of ACO (**Table 2**). In addition, we checked correlation (**Table 3**) between EMT markers from samples of ACO and epithelial macrophage that we reported to be higher in ACO earlier (**Figure 5**). Notable correlations trend considering spearman r values were (a) a trend of moderate positive correlation between mesenchymal markers (vimentin and S100A4 positive basal cells) in the epithelium and FEV_1_/FVC ratio (spearman *r*, 0.3357 and 0.4825, respectively); (b) a trend of moderate positive correlations between smoking history and mesenchymal markers (Vimentin and S100A4 positive basal cells with the spearman *r* of 0.3825 and 0.2947, respectively); (c) a trend of weak positive correlation between E-cadherin and epithelial macrophages (**Figure 5A**) (d) significant and moderate positive correlation between epithelial macrophage and epithelial-mesenchymal markers (S100A4 and vimentin positive basal cells with the spearman *r* of 0.5245 and 0.6294; *P* = 0.0161 and 0.0420 respectively) (**Figures 5C, D**). We dichotomized the ICS-treated and nontreated patients with ACO to see if there is any effect on the EMT markers (**Figure 6**) We noted a marked decrease in vimentin (*P <*0.05) and S100A4 (*P <*0.001) basal cells in ICS treated patients as compared to patients without ICS treatment. Furthermore, Vimentin and S100A4-positive RBM cells also appeared to be lower in ICS-treated patients as compared to patients without ICS treatment. Interestingly, the degree of fragmentation tended to be higher in ICS treated patients as compared to patients without ICS treatment.

**Table 3:** Spearman correlation analysis.

Parameters	FEV_1_/FVC		Smoking history	
	Spearman r	*P* value	Spearman r	*P* value
Percent staining E-cadherin	-0.04196	0.452	0.06667	0.4189
Percent staining N-cadherin	-0.2273	0.2517	-0.1507	0.3283
Vimentin-positive basal cells	0.3357	0.1434	0.3825	0.1094
Vimentin-positive RBM cells	-0.04196	0.452	0.1474	0.3229
S100A4 positive basal cells	0.4825	0.0577	0.2947	0.1748
S100A4 positive RBM cells	-0.2448	0.2217	0.04211	0.4496
Degree of fragments	-0.2238	0.2426	0.1228	0.3513

RBM, reticular basement membrane, FEV_1,_ forced expiratory volume in 1 second; FVC, forced vital capacity.

### Immunoblot protein expression of EMT markers

**Figure 7:**
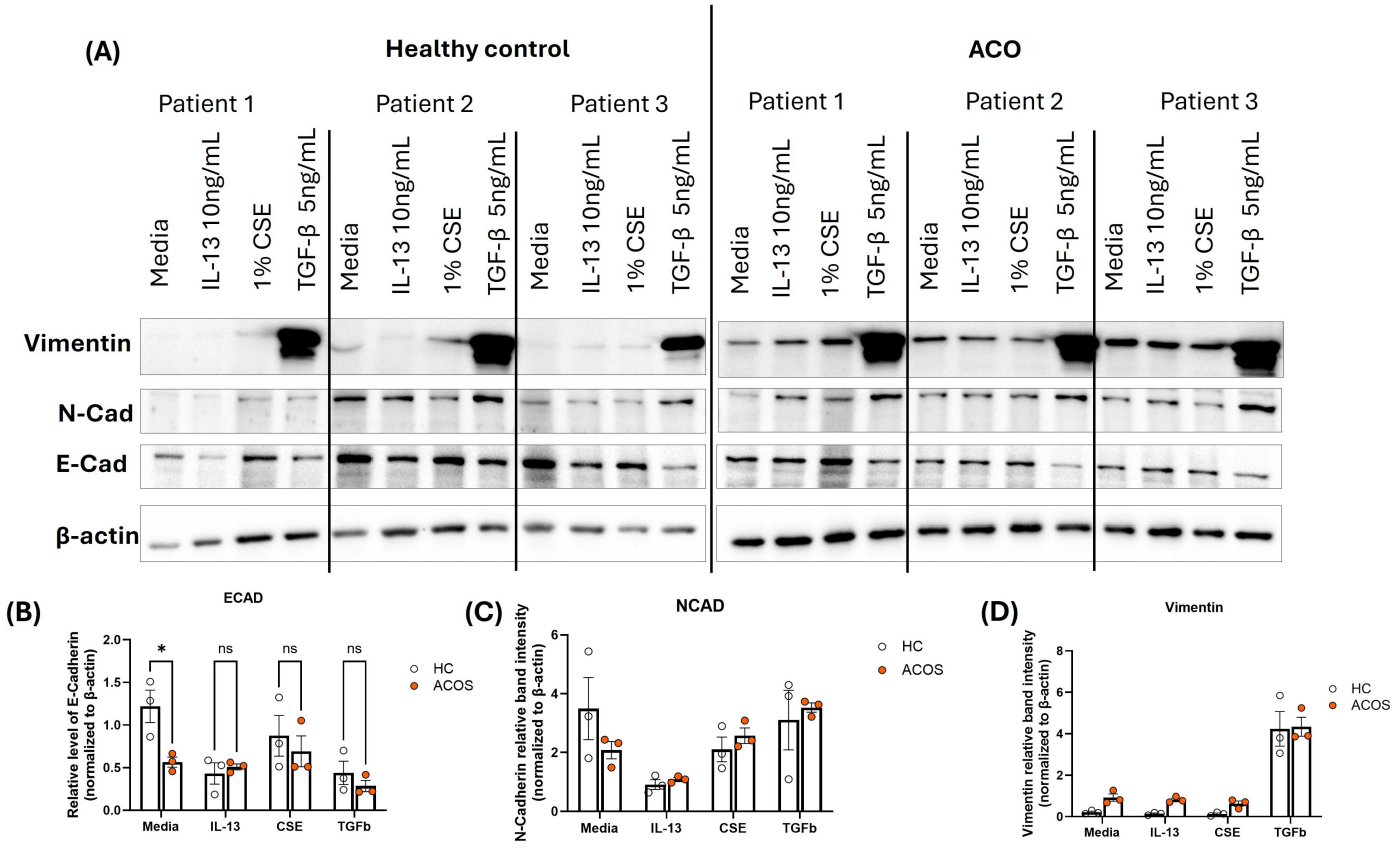
Protein expression of EMT markers in the ACOS airway epithelium. **(A)**. Protein lysate from different treatments from healthy and ACO pBEC ALIs were used to immunoblot against E-Cadherin, N-Cadherin and Vimentin. β-actin was used as the loading control (n=3). **(B–D)**. The band intensity was measured and normalized to the β-actin level and the fold expression of **(A)**. E-Cadherin **(B)**. N-Cadherin and **(C)**. Vimentin was graphed. Two-group comparisons were analyzed with an unpaired t-test. *P ≤ 0.05.

**Figure 8:**
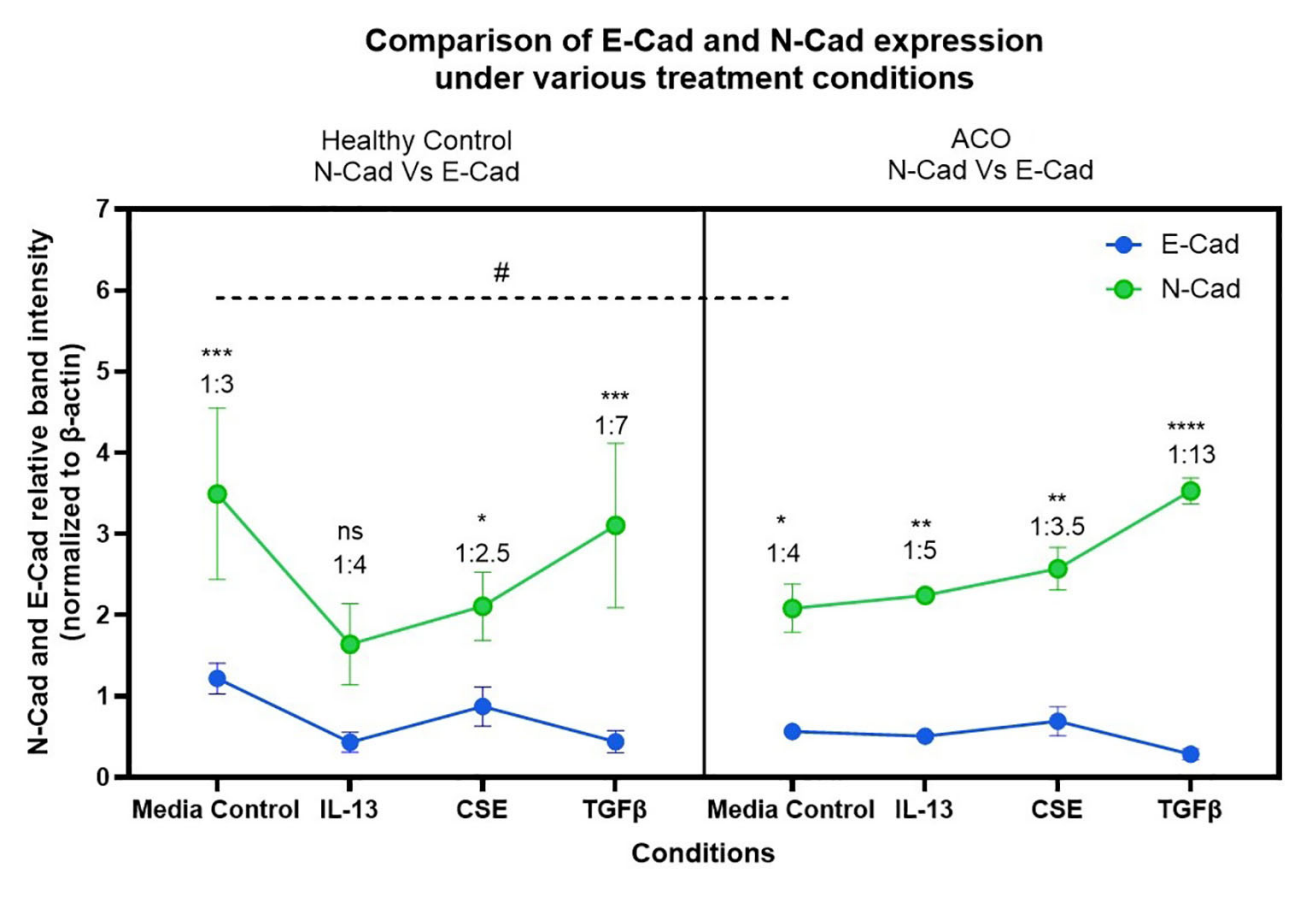
Relative expression changes of N-cadherin and E-cadherin in human-derived lung primary epithelial cells (HC and ACO patients) post induction with Th-2 cytokine IL-13, CSE and EMT activator TGF-β. ^#^ p<0.05, *p<0.05, **p<0.01, ***p<0.001, ****p<0.0001.

The protein expression of EMT markers; E-cadherin, N-cadherin and Vimentin was measured by performing immunoblot. β-Actin was used as the internal control. The intensity of protein bands was measured, and the fold expression was graphed. Our results show that E-cadherin protein expression is significantly low in the ACO group compared to the healthy group at baseline level (**Figures 7A, B**). IL-13 and TGF-β drastically reduce the expression of E-cadherin in the healthy group but not in ACOS. The expression of N-cadherin protein level was similar between ACO and healthy across all treatments (**Figures 7A, C**). Interestingly, the expression of vimentin protein level was significantly higher in ACOS at baseline (p≤ 0.05) as well as with IL-13 and 1% CSE treatment (**Figures 7A, D**). We also compared relative expression change of N-cadherin and E-cadherin in response to treatment (**Figure 8**). Although similar in ratios, N-cadherin expression in HC media control was higher than in ACOS (#*P*<0.05) media control. Within-group variability was more prominent in HC N-cadherin expression across the treatment groups than in ACOS patients. There were higher significant changes in expression (p<0.05, p<0.01, p<0.001, p<0.0001) between N-cadherin and E-cadherin in ACOS than in HC. The ratio change between N-cadherin and E-cadherin was most significant in the ACOS patients induced with TGF-β, double that of HC.

## Discussion

This exploratory EBB study unequivocally demonstrated a marked decrease in epithelial E-cadherin and a trend of increased mesenchymal marker N-cadherin in the airway epithelium of patients with ACO. This study also demonstrated a trend of high vimentin and S100A4-positive basal and RBM cells in ACO, and an RBM with the elevated degree of fragmentation as represented by the cleft formation in ACO as compared with HC and asthma. Similar changes were observed with western blots in response to Th-2 cytokine IL-13, CSE and EMT activator TGF-β. These preliminary findings could provide new insights into ACO research and support our hypothesis of an active EMT process in the airways of patients with ACO.

Although the EMT process is considered to be an active process in COPD, in ACO there are no reports on EMT yet. It is suggested that following injury, adhesion junction modifications are critical for the structural rearrangement of the airway epithelial cell to allow spreading, migration, and subsequent epithelial proliferation on the provisional fibrin extracellular matrix (ECM). One of the important features of EMT is a decrease in epithelial markers and (3) and a concomitant increase or acquisition of mesenchymal markers with associated RBM fragmentation. Both E- and N-cadherins are traditional type I cadherins (30). Cadherin cytoplasmic tails bind to β-catenin, which in turn binds to α-catenin, forming the cadherin-catenin adhesion complex which maintains tissue stability and dynamic cell movements. By influencing a wide variety of signalling pathways, E-cadherin plays a critical role in both the preservation of the epithelial phenotype and the maintenance of tissue homoeostasis. Our observation of decreased epithelial E-cadherin and increased N-cadherin expression in patients with ACO is, therefore, suggestive of an EMT process in these patients.

In addition, other widely used mesenchymal markers vimentin and S100A4 were also reported to be increased in EMT (8, 31). Vimentin is found in all mesenchymal cells and is at the heart of EMT-mediated metastasis (32). Vimentin can induce cell migration during EMT by forming cell processes, decreasing cell adhesion, and increasing cell migration ability. In fact, in previous research from our laboratory, enhanced vimentin-positive cells were reported in smoking-associated COPD patients. In the context of EMT, S100A4 is also regarded as a typical mesenchymal marker. Its biological roles include the promotion of cell motility, invasion, extracellular matrix (ECM) remodelling, autophagy, and angiogenesis (33). Elevated vimentin and S100A4-postive basal and RBM cells in patients with ACO thus are an indication of the fact that the cells are undergoing transition to a mesenchymal phenotype and attaining more migratory phenotype.

The increased RBM fragmentation is quite novel finding in the large airway tissues of patients with ACO and is a tissue hallmark of active EMT. During EMT, transitioning epithelial cells gain a migratory potential and digest their way through basement collagen into the subepithelial lamina propria to become fibroblasts (8, 34). In addition, fragmentation or rupture was also reported in the kidney tubular RBM of patients with acute cellular rejection (35). In contrast to increased fragmentation in the ACO, which is suggestive of a prominent COPD component in ACO, we found low fragmentations in asthma, which were essentially similar to HC. Indeed, the fragmentation results were consistent with our prior report, in which we observed much fewer fragmentations than those seen in COPD and were comparable to those seen in HC (8).

EMT is indeed an active process in both small and large airways of smokers and COPD patients, with consequences for lung physiological parameters in these patients (36). The current study data especially related to COPD, nearly mimic the previously reported data from my laboratory on E-cadherin, N-cadherin, and vimentin (8, 37). The results of EMT markers in asthma especially the ratio of E and N-Cadherins suggests an active EMT process in these patients as indicated in many studies reported in relation to EMT in asthma based on simulated epithelial cells, *in-vivo* animal model data, and house duct mice induced lungs (38–40), although EMT in asthma remains a subject of debate as no human tissue evidence is available including the hallmark of RBM fragmentation. We believe EMT is not part of the asthma pathology, but you may see some epithelial cell integrity compromised (41).

Our preliminary report on inflammatory cell profile in the large airway of patients with ACO indicated dominant macrophages in the epithelium, RBM, and LP. Macrophages were shown to induce EMT, promoting metastasis of lung cancer cells through COX-2/PGE2/β-catenin pathways (42). Our cell culture/western blot data also shows role of inflammation in driving EMT changes, but more work is needed. Indeed, we noticed a positive correlation between significant and moderate positive correlation between epithelial macrophage and epithelial-mesenchymal markers. Recently we reported increased vascularity in the RBM of ACO patients, characterizing type III EMT in these patients, which is recognised as producing highly dangerous pre-cancerous stroma under the epithelium. Active type 3 EMT is considered as precursor to malignant conditions and metastasis (13, 43).

Intervention with ICS could play an important role in reducing EMT, a critical pathology of chronic respiratory diseases, particularly COPD (43, 44). Previously in a clinical study, we reported regression of EMT biomarkers in bronchial biopsies of patients with COPD who were on ICS treatment (44). In line with our previous findings, we noted a dampening effect of ICS treatment on mesenchymal markers in ACO patients, suggesting the prevention of epithelial phenotype transitioning to mesenchymal cells.

The current study findings are novel and should be considered as preliminary. Clinical samples from ACO patients are very rare. We expect reasonable criticism on the sample size used in this study; however, we also noticed a robust difference between groups. Furthermore, our study lacks mechanistic data elucidating the underlying biological processes associated with our histopathological examination. Integrating data from mechanistic study will provide a more holistic understanding of the disease mechanisms. We also acknowledge the lack of documented environmental exposures, comorbidities, medication adherence, and lifestyle factors, may have some influence on outcomes. Future studies including these factors will be informative.

Although further investigations are required, the present study contributes to a better understanding on an active EMT process in patients with ACO. In the absence of sufficient literature on EMT in patients with ACO, we believe that our research findings will be informative not only to better understand the ACO process, but also the patient management by targeting EMT, a novel and promising platform, with ICS.

## Data Availability

The raw data supporting the conclusions of this article will be made available by the authors, without undue reservation.
